# Developments in NEW triad research

**DOI:** 10.18632/aging.204062

**Published:** 2022-05-04

**Authors:** Shawn N. Whitehead, Vladimir Hachinski, Alexander Levit

**Affiliations:** 1 Vulnerable Brain Laboratory, Department of Anatomy and Cell Biology, Schulich School of Medicine and Dentistry, University of Western Ontario, London, ON N6A 5C1, Canada; 2 Department of Clinical Neurological Sciences, University Hospital, University of Western Ontario, London, ON N6A 5A5, Canada; 3 Department of Psychiatry, University of British Columbia, Vancouver, BC V6T 2A1, Canada

**Keywords:** dementia, executive function, white matter, neurovascular unit, blood-brain barrier

In 2020, we offered Neurovascular unit (NVU) dysregulation, Executive dysfunction, and White matter disease as a triad of understudied pathologies that are central to the most common and often comorbid neurodegenerative diseases, vascular cognitive impairment (VCI) and Alzheimer disease (AD) [1]. We considered it a helpful construct as it cuts across the cellular, neuropsychiatric, and histological/radiological domains of study. Since then, we have been calling it the NEW Triad. Several key studies have advanced this theoretical construct further.

In a study of healthy aging by Verheggen et al., dynamic contrast-enhanced (DCE) MRI captured a greater propensity for blood-brain barrier (BBB) leakage in regions of higher-order function (bilateral orbitofrontal and cingulate cortices), that is executive function. A greater leakage rate was seen in white matter than in gray matter, though not statistically significant [2]. Kerkholfs et al., demonstrated that in patients with cerebral small vessel disease, leakage volume in both radiologically normal appearing white matter and white matter hyperintensities correlated with cognitive decline over 2 years of follow-up, particularly in the domain of executive function [3]. These studies support prior findings that these brain regions are inherently more vulnerable to vascular injury but moreover, identify DCE-MRI as a potentially important tool in both research and clinical settings. Future studies can utilize DCE-MRI to evaluate mechanisms of cognitive protection in interventions that target either the NVU or systemic vascular risk factors.

Complementing these imaging tools, Hussain et al., summarize the many potential serum biomarkers that could be used to detect BBB leakage [4]. Several of these biomarkers are proteins released from cells of the NVU. Serum biomarkers will be important in the research and development setting as they allow for *in vivo* corroboration of neuroimaging, such as DCE-MRI studies. More importantly, in the clinical setting, serum biomarkers of neurodegenerative processes would offer much needed correlates for diagnosis and monitoring of treatment response. In many settings, serum biomarkers will continue to be far more accessible to researchers and patients than MRI (and certainly more accessible than specialized MRI protocols such as DCE-MRI).

A question that largely remains outstanding is the causal relationship between the NEW triad and the various brain parenchymal proteinopathies, namely of amyloid and tau, that are associated with neurodegenerative syndromes such as AD. Given that the NEW triad has been captured even in clinically healthy aging, that is, it precedes clinical manifestations, it is tempting to pursue the hypothesis that NEW triad pathology is causal. Variations in NEW triad severity may ultimately lead to the difference between healthy neurological aging and decompensation of CNS homeostasis with subsequent neurodegenerative disease. However, an important alternative hypothesis is that toxic protein accumulation in AD is instead the ‘upstream’ process that leads to abluminal injury of the NVU, which then develops the NEW triad [1]. Lastly, a hypothesis of confounding should be considered, in which the NEW triad and neurodegenerative proteinopathies are both independently caused by a confounding factor, such as microglial [1] or glymphatic pathology [5].

While it would be prudent to delineate causality, the NEW triad as a theoretical construct is hoped not to complicate a field that is already fraught with many unresolved hypotheses. Rather, as vascular risk factors and glial cells are likely more accessible to therapeutic intervention, we hope that a focus on the NEW triad would expand the research of treatment options, including novel therapeutic targets. Firstly, the repurposing and optimization of well-established treatments for hypertension, diabetes, and dyslipidemia offer many opportunities for protection of the NVU from a luminal angle [6]. Soto-Rojas et al., have also outlined novel treatments including new drug delivery systems that could protect the NVU from the abluminal angle, such as nanoparticles, liposomes, and so-called molecular trojan horses that rely on receptor-mediated transcytosis [6]. Empiric therapeutic studies in AD (or any neurodegenerative conditions associated with the NEW triad) could be criticized as putting the cart before the horse, as the pathophysiology of these conditions is not yet well understood. However, in the face of a potential public health crisis perpetuated by an overburdened long-term care system, jumping to studies of intervention is warranted. It is important to recall that executive dysfunction is the largest predictor for loss of functional independence in the context of dementia, and thus a priority for preserving patient quality of life and investment of healthcare resources [1].
